# Association between Dietary Fiber Intake and Mortality among Colorectal Cancer Survivors: Results from the Newfoundland Familial Colorectal Cancer Cohort Study and a Meta-Analysis of Prospective Studies

**DOI:** 10.3390/cancers14153801

**Published:** 2022-08-04

**Authors:** Jing Zhao, Yun Zhu, Meizhi Du, Yu Wang, Jillian Vallis, Patrick S. Parfrey, John R. Mclaughlin, Xiuying Qi, Peizhong Peter Wang

**Affiliations:** 1 Department of Epidemiology and Biostatistics, School of Public Health, Tianjin Medical University, Tianjin 300070, China; 2 Division of Community Health and Humanities, Faculty of Medicine, Memorial University of Newfoundland, St. John’s, NL A1B 3V6, Canada; 3 Clinical Epidemiology Unit, Faculty of Medicine, Memorial University of Newfoundland, St. John’s, NL A1B 3V6, Canada; 4 Dalla Lana School of Public Health, University of Toronto, Toronto, ON M5T 3M7, Canada

**Keywords:** colorectal cancer, dietary fiber intake, all-cause mortality, CRC-specific mortality

## Abstract

**Simple Summary:**

High dietary fiber intake has been linked to a lower risk of Colorectal cancer (CRC), but the role of dietary fiber in CRC survival is understudied. We examined dietary fiber intake for its relevance to CRC survival in a cohort of 504 CRC patients and a meta-analysis including results from four prospective cohort studies. We found that high dietary fiber intake was negatively correlated with all-cause mortality and CRC-specific mortality among CRC survivors. These new findings support the protective effect of dietary fiber on CRC survival. By enhancing fiber intake, this research may contribute to the development of novel therapies that add to our armamentarium for CRC.

**Abstract:**

We examined dietary fiber intake for its relevance to Colorectal cancer (CRC) survival in a cohort of CRC patients and a meta-analysis including results from four prospective cohort studies. We analyzed 504 CRC patients enrolled in the Newfoundland Familial Colorectal Cancer Study (NFCCS) who were newly diagnosed with CRC between 1999 and 2003. Follow-up for deaths was through April 2010. All participants completed a self-administered food frequency questionnaire to evaluate their dietary intakes one year before diagnosis. Multivariable Cox proportional hazard models were used to explore the associations of dietary fiber intake with all-cause mortality and CRC-specific mortality. In the meta-analysis, we identified prospective cohort studies published between January 1991 and December 2021 by searching PubMed, EMBASE, and Cochrane Library. Fixed-effects or random-effects models were used to combine the study-specific hazard ratio (HR) from our original analysis and three other cohorts. In the NFCCS, we found that CRC patients with the second quartile of dietary fiber intake had a 42% lower risk of all-cause mortality (HR: 0.58, 95% CI: 0.35–0.98) and 58% lower risk of CRC-specific mortality (HR: 0.42, 95% CI: 0.21–0.87) compared with those with the lowest quartile. In the meta-analysis, a similar inverse association between dietary fiber and total mortality was detected among CRC patients; each 10 g/day increase in dietary fiber intake was associated with a 16% decreased risk of total mortality. The dose–response meta-analysis showed a linear relationship between dietary fiber intake and all-cause mortality, with no sign of a plateau. For CRC-specific mortality, intriguingly, the benefit associated with increasing dietary fiber intake achieved its maximum at approximately 22 g/day, and no further reduction in CRC-specific mortality was observed beyond this intake level. Our results suggest that high dietary fiber intake may be associated with prolonged survival among CRC patients. Our findings add to the sparse literature on the role of dietary fiber in CRC survival.

## 1. Introduction

Colorectal cancer (CRC) is the third most common cancer and the second most common cause of cancer death in Canada and worldwide [1,2]. It is estimated that approximately 1 in 37 (3%) Canadians died from CRC in 2021 [1]. Although CRC survival has consistently increased for a decade with improvements in early diagnosis and treatment of the disease [3], fewer than 20% of patients diagnosed with metastatic CRC survive beyond 5 years from diagnosis. Many CRC survivors actively seek dietary counseling to promote their cancer treatment and rehabilitation [4]. However, since studies on dietary factors in relation to CRC survival are sparse, most dietary recommendations for CRC survivors are mainly based on incidence studies [5,6]. Therefore, there is a need for more research on diet and prognosis among CRC survivors whose potential health benefits from optimal diet is of public health importance for policies and plans to support health efforts. 

Since the middle of the 20th century, much research has been done to better understand the physicochemical properties of dietary fiber and its benefits to physiology and metabolism [7,8]. Although high dietary fiber intake has been suggested to protect against CRC development [9,10], its effect on the survival of CRC is largely unknown. The only five available studies on the relationship between dietary fiber intake and survival of CRC patients have yielded inconclusive results. Song et al. [11] found that patients with a higher fiber intake had a lower rate of CRC-specific mortality and all-cause mortality, whereas another three studies yielded null results [12,13,14]. Counter-intuitively, a study published in 1989 reported higher risks of all-cause mortality associated with higher fiber intake among CRC patients [15]. Nevertheless, these published reports were mainly performed in the United States, France, and Western Europe, but none were in Canada. A recent meta-analysis demonstrated that post-diagnostic rather than pre-diagnostic intake of fiber was significantly associated with CRC-specific mortality (HR: 0.54, 95% CIs: 0.35–0.84) [16]. However, the evidence from the current body of original research and meta-analyses is insufficient to draw a consistent conclusion. The relationship between dietary fiber intake and CRC survival has yet to be resolved. 

Therefore, we examined the association of dietary fiber intake with all-cause mortality and CRC-specific mortality within the context of the Newfoundland Familial Colorectal Cancer Study. Additionally, we quantitatively gathered current evidence regarding the fiber–CRC survival association in a meta-analysis, including results from the previous studies and those from ours.

## 2. Materials and Methods

### 2.1. The Newfoundland Familial Colorectal Cancer Study

#### 2.1.1. Study Population

The Newfoundland Familial Colorectal Cancer Study (NFCCS) has been described in detail in other published articles [17,18,19,20,21]. In brief, eligible participants were newly diagnosed CRC patients identified from the Newfoundland Colorectal Cancer Registry (NFCCR) between 1999 and 2003 and aged between 20 and 75 years. Exclusion criteria for this analysis included: (1) patients with unknown vital status at the end of follow-up (*n* = 1), (2) patients with insufficient information on dietary fiber intake or other crucial prognostic risk factors (*n* = 206), and (3) those with a reported total calorie intake of more than 2.5% or less (*n* = 26; 870 and 4330 kcal/d for male, 1010 and 5050 kcal/d for female, respectively). If a patient died before he/she could complete the questionnaires, a family proxy who had lived with the patient was invited to participate. As a result, the final cohort consisted of 504 consenting participants (68.7% of all eligible CRC patients from the NFCCR).

Written informed consent was signed by all consenting participants, and the study was conducted with the approval of the Health Research Ethics Authority, Memorial University of Newfoundland, following the Declaration of Helsinki.

#### 2.1.2. Diet assessment and Baseline Information Collection

Participants completed and returned a detailed food frequency questionnaire (FFQ), personal history questionnaire (PHQ), and family history questionnaire (FHQ).

The self-administrated FFQ was used to evaluate dietary fiber intake from one year before CRC diagnosis [20,21,22]. The FFQ applied in the present study included 169 food items/beverages and had been previously validated within the Newfoundland population [23]. Participants were asked about their consumption frequency and usual portion size of 169 food items. Nutrients and total energy intake were computed by multiplying the consumption frequency of each food by the nutrient content of the portion size based on the composition values in the 2005 Canadian Nutrition file [18,24,25]. 

The PHQ and FHQ were utilized to collect detailed information such as demographics (e.g., age, sex, education attainment), personal lifestyle (e.g., physical activity, alcohol and tobacco use), bowel screening history, and family history of cancer. The BRAF V600E mutation and the microsatellite instability (MSI) status in tumor tissue were assessed using standard protocols [21,26,27].

#### 2.1.3. Study Outcomes

The cohort was followed until death or April 2010, whichever came first. The vital status of participants was determined from questionnaires, local newspapers, death certificates, autopsy, pathology, radiology, surgical reports, as well as physicians’ notes. Additional data were ascertained from the Dr. H Bliss Murphy Cancer Care Foundation and Statistics Canada [19]. The cause of death of 159 deceased patients in this cohort was classified according to the ICD codes for underlying or contributing causes of death [28]. Participants who did not have events of interest by the end of the follow-up were censored at the last tracking date.

#### 2.1.4. Statistical Analysis

Log-rank test was used to compare the survival distribution of baseline characteristics in each group. Multivariable Cox regression models were used to calculate the hazard ratios (HRs) and 95% confidence intervals (CIs) for the association between a defined endpoint event and the quartile of dietary fiber intake before diagnosis, using the first quartile as a reference. Covariates were retained in the final model if they changed the effect estimates by over 10% or with a *p*-value less than 0.1 in the log-rank test. These covariates included sex, age at diagnosis, stage at diagnosis, marital status, MSI status, BRAF mutation status, reported chemoradiotherapy, and total energy intake. The proportional hazards assumption was verified by testing the significance of an interaction term between the explanatory variable and the logarithm of follow-up time for each covariate. In our analyses, bilateral *p*-values less than 0.05 indicated a significant difference. Data analyses and management were performed using SAS software version 9.4 (SAS Institute Inc., Cary, NC, USA).

### 2.2. Meta-Analysis

#### 2.2.1. Literature Search Strategy and Study Selection

Two authors independently used medical subject heading (MeSH) terms and free texts to conduct a systematic search of studies published from January 1991 to December 2021 in PubMed, EMBASE, and Cochrane Library. After several pre-searches, we determined the specific search strategy (Table S1). In order to identify additional relevant studies, we manually searched the bibliographies of retrieved reviews and articles. Subsequently, we manually reviewed the titles, abstracts, full-text articles, and references after eliminating potential duplicates. The inclusion and exclusion criteria for the studies are shown in Table S2.

#### 2.2.2. Data Extraction

The data were collected independently by two authors; any discrepancies were settled by discussion or by a third reviewer until a consensus was reached. The systematic review followed the recommendations of the Preferred Reporting Items for Systematic Reviews and Meta-Analyses (PRISMA) [29]. The protocol has not been registered.

The Newcastle–Ottawa scale was used to assess the risk of bias in the included studies and rank them in order (a score of 8 to 9 indicates high quality, 5 to 7 indicates medium quality, and a score of 4 or less indicates low quality) [30]. The detailed scores are provided in Table S3.

#### 2.2.3. Statistical Analysis

We pooled the HRs and their corresponding 95% CIs in a fixed-effects or random-effects model by combining the multivariable-adjusted HR of the highest compared with the lowest dietary fiber intake. When studies reported results separately by sex, we included them independently within the pooled estimate. Finally, the pooled HRs and 95% CIs were displayed in forest plots.

To examine the potential dose–response relationships of fiber consumption with all-cause and CRC-specific mortality, we applied a two-stage random-effects dose–response meta-analysis [31]. The pooled HRs and 95% CIs per 10 g/day increase in fiber were illustrated by forest plots. Restricted cubic splines with three knots fixed at the 10th, 50th, and 90th percentiles of dietary fiber intake were employed to assess potentially nonlinear dose–response associations [32,33,34]. Finally, the derived curves were combined using a multivariable meta-analysis [35,36].

Heterogeneity across studies was examined by Cochran’s Q test and I^2^ statistic [37,38]. I^2^ value greater than 50% suggested substantial heterogeneity [38,39]. Publication bias was statistically assessed by Begg’s funnel plot and Egger’s test [40].

This meta-analysis was performed according to the Meta-analyses Of Observational Studies in Epidemiology guidelines (MOOSE) [41]. A two-sided significance level was set at alpha = 0.05. All analyses were conducted using Stata version 15.0 (StataCorp LLC, College Station, TX, USA).

## 3. Results

### 3.1. The Newfoundland Familial Colorectal Cancer Cohort Study

#### 3.1.1. Patient Characteristics

By the end of the follow-up, a total of 159 (31.5%) patients died (median follow-up time, 6.4; minimum, 1.3 years; maximum, 10.9 years), and 30 patients experienced CRC recurrence or metastasis. The average age of the study population was 60.9 ± 9.0 years, of which 65.1% had tumors in the colon subsite. In the univariate analyses, young age at diagnosis, female gender, early stage at diagnosis (I/II), and MSI-H tumors were negatively correlated with all-cause mortality (Table 1). Except for total energy intake, the salient traits were largely comparable between the subjects included and those excluded due to missing responses on diet/vital status or unreliable total energy intake (Table S4).

#### 3.1.2. Dietary Fiber Intake and All-Cause Mortality

The risk of all-cause mortality among CRC patients decreased by 42% for subjects in the second quartile compared with those in the lowest quartile of dietary fiber consumption (Table 2). This inverse association was limited to patients diagnosed with colon cancer only.

Prior research has revealed gender differences in eating habits, and dietary fiber intake is generally lower in males than in females. We, therefore, investigated whether the dietary fiber–CRC survival association varied by sex. However, no drastic gender differences in the association between dietary fiber and all-cause mortality were observed.

#### 3.1.3. Dietary Fiber Intake and CRC-Specific Mortality

Compared with the lowest quartile, the second quartile of dietary fiber intake was associated with a decreased risk of CRC-specific mortality (HR: 0.42, 95% CI: 0.21–0.87) (Table 2). The protective effect of fiber consumption against CRC-specific mortality was only evident in males (vs. females) (HR: 0.28, 95% CI: 0.09–0.86) and colon cancer (vs. rectum cancer) patients (HR: 0.31, 95% CI: 0.12–0.82).

### 3.2. Meta-Analysis

#### 3.2.1. Study Selection and Characteristics

**Figure 1:**
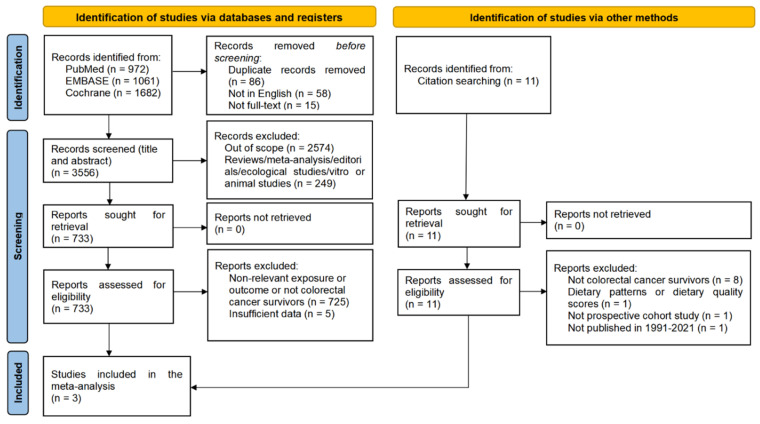
Flow chart of study selection.

Among the 3715 publications assessed, three studies [11,12,13] had examined the influence of dietary fiber intake on mortality in CRC patients (Figure 1). A total of 2240 deaths accrued in 6016 CRC patients were included in the meta-analysis of dietary fiber in relation to all-cause and CRC-specific mortality (including three published cohort studies and the current research) (Table 3). The quality of these observational studies was generally moderate to good (Table S3).

#### 3.2.2. Association between Dietary Fiber Intake and All-Cause Mortality

**Figure 2:**
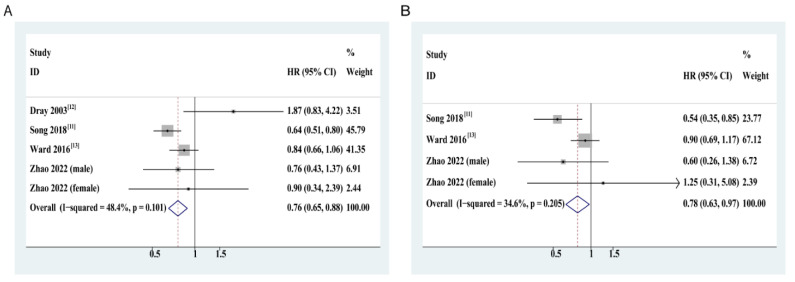
Summary forest plots of hazard ratios (HRs) with 95% confidence intervals (CIs) of mortality for highest dietary fiber intake relative to lowest. (**A**) All-cause mortality; (**B**) CRC-specific mortality. Abbreviations: CRC, colorectal cancer; HR, hazard ratio; CI, confidence interval. Squares indicate study-specific HR estimates (the size of the square reflects the study-specific statistical weight, i.e., the inverse of the variance); horizontal lines indicate the 95% CI; diamonds indicate the pooled HRs with their 95% CI. Study-specific HR estimates were pooled using a fixed-effects model.

We observed a significantly negative correlation between dietary fiber intake and all-cause mortality among CRC survivors. The pooled HRs and 95% CI of all-cause mortality when comparing persons in the top category of dietary fiber intake with those in the bottom category was 0.76 (95% CI: 0.65–0.88) (Figure 2A). The funnel plot was symmetrical with one small study outlier (Figure S1). Both Begg’s (*p* = 0.46) and Egger’s (*p* = 0.29) tests were non-significant (Figure S2). The heterogeneity detected among studies was medium and not statistically significant (I^2^ = 48.4%; *p* = 0.101).

**Figure 3:**
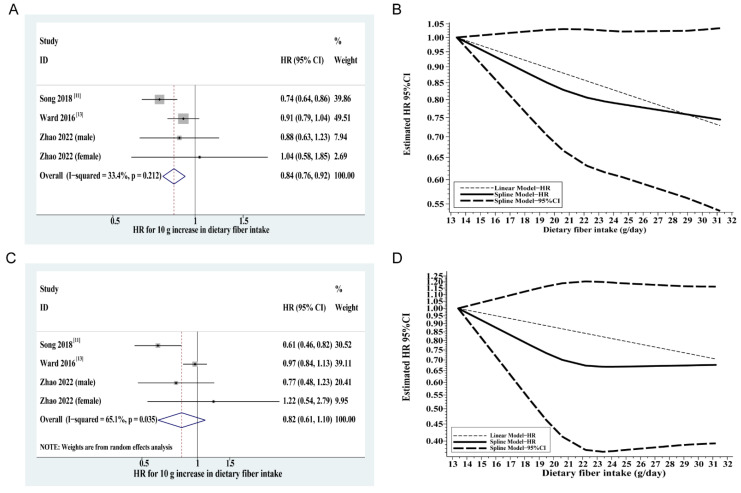
Linear and nonlinear dose–response meta-analyses of dietary fiber and mortality. (**A**) Linear dose–response meta-analyses per 10 g/day increase in dietary fiber intake for all-cause mortality; (**B**) Linear and nonlinear dose–response curve for all-cause mortality; (**C**) Linear dose–response meta-analyses per 10 g/day increase in dietary fiber intake for CRC-specific mortality; (**D**) Linear and nonlinear dose–response curve for CRC-specific mortality. Abbreviations: CRC, colorectal cancer; HR, hazard ratio; CI, confidence interval.

In the dose–response meta-analysis, there was no evidence of nonlinearity in the relationship between dietary fiber intake and all-cause mortality with no sign of a plateau within the available data (*p* nonlinearity = 0.32) (Figure 3B). The risk of all-cause mortality among CRC patients decreased by 16% for each 10 g/day increase in dietary fiber intake (HR: 0.84, 95% CI: 0.76–0.92); the heterogeneity was not evident (I^2^ = 33.4%, *p* = 0.212) (Figure 3A).

#### 3.2.3. Association between Dietary Fiber Intake and CRC-Specific Mortality

Compared with the lowest quartile of dietary fiber intake, the highest quartile of dietary fiber intake was associated with a 22% (HR: 0.78, 95% CI: 0.63–0.97) decrease in the risk of CRC-specific mortality (Figure 2B). Egger’s test indicated no evident publication bias (*p* = 0.75). Overall, the level of heterogeneity was low (I^2^ = 34.6%) and was not statistically significant (*p* = 0.205). In the dose–response meta-analysis, intriguingly, we observed a threshold in the dose–response curve with no further reduction in the risk of CRC-specific mortality (e.g., plateau effect) beyond 22 g/day intakes of dietary fiber (Figure 3D). The pooled HR of CRC-specific mortality for each 10 g/day increase in fiber intake was not significant (HR: 0.82, 95% CI: 0.61-1.10, heterogeneirty: I^2^=65.1%, *p* =0.035) (Figure 3C).

## 4. Discussion

Our analysis of the NFCCS found a beneficial influence of dietary fiber on CRC survival, although our data showed a J-shaped relationship, with the strongest correlation observed among patients in the second quartile of dietary fiber intake. Only five studies have thus far investigated the relationship between dietary fiber intake and survival after CRC diagnosis. Similar to our findings, in the Nurses’ Health Study and the Health Professionals Follow-up Study, higher dietary fiber intake was significantly associated with lower mortality among CRC patients. For each 5 g/day increase in fiber intake, the risk of all-cause mortality and CRC-specific mortality decreased by 14% (HR: 0.86, 95% CI: 0.79–0.93) and 22% (HR: 0.78, 95% CI: 0.65–0.93), respectively [11]. The beneficial relationship appeared to differ by the sources of fiber, with fiber from cereals showing the strongest association (all-cause mortality: cereal fiber: HR per 5 g/day increment, 0.78, 95% CI, 0.68–0.90; vegetable fiber: HR, 0.83, 95% CI, 0.72–0.96; fruit fiber: HR, 0.92, 95% CI, 0.78–1.08) [11]. The results of the other three studies conducted in France, Europe (ten countries), and the United States indicated a null association between dietary fiber intake and CRC survival [12,13,14]. In a study of Caucasian CRC patients in Utah, the United States, higher pre-diagnostic fiber intake was counter-intuitively associated with poor CRC survival rates. However, about three-quarters of the study participants in the Utah study belonged to members of the Church of Jesus Christ of Latter-day Saints (LDS), whose lifestyle prohibited the use of alcohol, coffee, or tobacco [15]. The unique population characteristics may impact the comparability and generalizability potential of the findings. Since the Utah study was published over the last 30-plus years, it was excluded from the subsequent meta-analysis. Beyond population difference, differences in dietary assessment tools (FFQs vs. other tools), range of intake level, chance, or the potential for confounding effects of important prognostic variables may also account for some of the conflicting evidence. For example, the MSI-high phenotype and somatic BRAF V600E mutation are strongly correlated with both diet and CRC survival; thus, it is important to adjust for these tumor phenotypes. However, tumor molecular phenotypes were not accounted for in previous studies.

Dietary fiber intake could affect mortality through several mechanisms. Dietary fiber may influence CRC survival by increasing fecal bulk, absorbing fecal carcinogens, binding secondary bile acids that might otherwise act as tumor promoters, taking them out of the digestive tract, and thereby decreasing interactions between colorectal tissue and carcinogens [42]. Fiber may also play a role in insulin sensitivity and metabolic regulation [7], which have been related to CRC prognosis. Moreover, short-chain fatty acids (SCFA) produced by fiber, such as butyrate and propionate, may help induce apoptosis in tumor cells [7,42,43,44,45,46,47].

In the current study, colon cancer, rather than rectal cancer, was found to be related to dietary fiber intake. Possible reasons for the observed heterogeneity include that (1) the average colonic transit time is longer than the rectal transit time, and (2) fermentable dietary fiber is mainly fermented and metabolized by gut bacteria in the colon rather than the rectum, which would lead to a stronger association with colon cancer. Admittedly, however, we cannot rule out that this is a matter of power.

When we combined the estimates from available prospective studies and ours in a meta-analysis, higher dietary fiber intake was associated with a lower risk of all-cause mortality in a linear manner. As for CRC-specific deaths, intake of approximately 22 g of fiber daily was associated with the lowest mortality, and higher intake was not related to additional risk reduction in mortality. The observed threshold effect is biologically plausible because dietary fiber, particularly soluble fiber, has limits in transport, metabolism, or storage, and their beneficial effects may be mediated by intestinal bacteria through fermentation that can be saturated. In a meta-analysis of dietary intake and CRC survival published in 2020, Hoang et al. [16] reported that fiber consumption was not related to either all-cause (HR: 0.84, 95% CI: 0.58–1.22) or CRC-specific mortality (HR: 0.72, 95% CI: 0.44–1.18). Hoang’s findings are apparently inconsistent with ours. It should be noted that our updated meta-analysis incorporated more studies (i.e., the NFCCS cohort) than the prior one, rendering us a greater power to reveal the association. Additionally, we performed a dose–response meta-analysis to delineate the relationship between mortality risk and exposure dose.

The strengths of this study included the prospective nature of the design, long-term follow-up (maximum 10.9 years), a moderately large sample size, and detailed collection of personal data and tumor molecular phenotype that allowed us to assess potential confounders. The present study had several limitations. First, the lack of data on post-diagnostic diet impeded us to assess the influence of diet and dietary alterations post-diagnosis on disease outcome, although previous research has shown minimal change in the amount of fiber consumption between pre-diagnosis and post-diagnosis among CRC patients [13,48]. Our analysis is based on the implicit hypothesis that pre-diagnostic dietary intake predicts a post-diagnostic dietary intake. Second, the measurement of diet is complex. Although the FFQ used in the present study contained a comprehensive list of foods and beverages and had been validated in previous research, measurement errors were inevitable in the estimates of nutrient consumption. Moreover, subjects with a high fiber diet may tend to overreport their intake, which may limit the ability to distinguish between the intakes of >22 g/day and 22 g/day. Thus, the possibility of modest benefits on mortality above 22 g/day of fiber intake could not be entirely ruled out [49]. Third, recall bias may skew the data since participants were asked to recall their dietary intakes from one year before diagnosis. Moreover, immortal time bias may influence the results, but this is minimized by using family proxies to enroll deceased cancer patients. Last, as a limitation shared by all meta-analyses on published articles, the cut-off level of dietary intake quantiles was generally inconsistent across studies; we may, therefore, have overestimated or underestimated the effects of fiber on CRC survival [16]. The between-study heterogeneity in our study may be attributed to the methodological or actual differences across studies.

## 5. Conclusions

In summary, the results from our cohort study and meta-analysis suggested that a high intake of dietary fiber was associated with improved CRC survival. Specifically, there was a linear relationship between dietary fiber intake and all-cause mortality. For CRC-specific mortality, the lowest risk was seen for around 22 g/day of fiber intake; above that level, however, the risk did not reduce further. As is always the case with novel epidemiologic findings, replication of this work is needed. If our findings were successfully replicated in other well-powered prospective studies, dietary fiber may serve as a potential target to improve therapeutic strategies for CRC patients. Future research could be undertaken to investigate the roles of different fiber sources in terms of CRC survival.

## Figures and Tables

**Table 1:** Demographical and clinicopathological characteristics of study population in the Newfoundland study (*n* = 504).

Characteristics	No. of Patients	No. of Deaths (%)	Univariate HR (95% CI) ^a^
Age at diagnosis (y) ^b^	60.9 ± 9.0	62.0 ± 8.9	1.02 (1.00–1.03)
Sex			
Male	306	106 (34.6)	1.00
Female	198	53 (26.8)	0.70 (0.50–0.98)
BMI (kg/m^2^)			
<25.0	140	43 (30.7)	1.00
25.0–29.9	203	70 (34.5)	1.06 (0.72–1.55)
≥30	146	41 (28.1)	0.91 (0.60–1.40)
Marital status			
Single	109	40 (36.7)	1.00
Married or living as married	395	119 (30.1)	0.85 (0.60–1.22)
Tumor location			
Colon	328	97 (29.6)	1.00
Rectum	176	62 (35.2)	1.19 (0.86–1.63)
Stage at diagnosis			
I/II	293	66 (22.5)	1.00
III/IV	211	93 (44.1)	2.36 (1.72–3.24)
T stage			
T1	25	5 (20.0)	1.00
T2	100	23 (23.0)	1.11 (0.42–2.93)
T3	308	107 (34.9)	1.74 (0.71–4.26)
T4	19	8 (42.1)	1.98 (0.64–6.07)
N stage			
NX	9	2 (22.2)	1.00
N0	264	66 (25.1)	1.29 (0.32–5.28)
N1	121	43 (35.5)	2.02 (0.49–8.35)
N2	55	30 (54.6)	3.74 (0.89–15.78)
M stage			
MX	221	56 (25.5)	1.00
M0	154	43 (27.9)	1.15 (0.77–1.71)
M1	39	31 (79.5)	6.84 (4.37–10.71)
Chemoradiotherapy			
No	100	38 (38.0)	1.00
Yes	404	121 (30.0)	1.36 (0.94–1.95)
MSI status			
MSS/MSI-L	423	146 (34.5)	1.00
MSI-H	55	4 (7.3)	0.17 (0.06–0.46)
BRAF mutation status			
Wild-type	411	133 (32.4)	1.00
V600E mutant	45	13 (28.9)	0.80 (0.45–1.41)
Smoking status			
Never smokers	138 (27.4)	36 (26.1)	1.00
Ever smokers	366 (72.6)	123 (33.6)	1.27 (0.87–1.84)
Total energy intake (kcal/d) ^b^	2455.3 ± 849.4	2491.53 ± 796.7	1.11 (0.96–1.27)

Abbreviations: HR, hazard ratio; CI, confidence; BMI, body mass index; MSI, microsatellite instable; MSI-H, microsatellite instability-high; MSS/MSI-L, microsatellite stable/microsatellite instability-low. For some variables, totals may not add up due to missing values. ^a^ Cox proportional hazard regression. ^b^ Continuous variables presented as mean ± SD (standard deviation).

**Table 2:** Associations of dietary fiber intake with all-cause mortality and CRC-specific mortality among CRC survivors.

	No. of Events ^a^ /No. at Risk	Quartiles of Dietary Fiber HR (95% CI) ^b^				*p*-Value for Trend ^c^
		Q1	Q2	Q3	Q4	
Mean (g/day)		14.17	19.74	24.15	30.35	
All-cause mortality						
All	159/504	1.00	0.58 (0.35–0.98)	0.93 (0.57–1.51)	0.80 (0.49–1.31)	0.716
Sex						
Male	106/306	1.00	0.65 (0.35–1.21)	0.83 (0.46–1.50)	0.76 (0.43–1.37)	0.451
Female	53/198	1.00	0.53 (0.19–1.46)	1.23 (0.49–3.11)	0.90 (0.34–2.39)	0.783
Anatomical subsite						
Colon cancer	97/328	1.00	0.44 (0.22–0.88)	0.76 (0.40–1.43)	0.55 (0.28–1.07)	0.264
Rectal cancer	62/176	1.00	0.78 (0.32–1.92)	1.37 (0.58–3.21)	1.59 (0.70–3.62)	0.187
CRC-specific mortality						
All	83/443	1.00	0.42 (0.21–0.87)	0.72 (0.36–1.43)	0.77 (0.39–1.52)	0.568
Sex						
Male	54/264	1.00	0.28 (0.09–0.86)	0.70 (0.32–1.56)	0.60 (0.26–1.38)	0.285
Female	29/179	1.00	0.73 (0.21–2.53)	0.93 (0.21–4.14)	1.25 (0.31–5.08)	0.677
Anatomical subsite						
Colon cancer	47/288	1.00	0.31 (0.12–0.82)	0.55 (0.21–1.45)	0.54 (0.20–1.51)	0.349
Rectal cancer	36/155	1.00	0.79 (0.26–2.42)	0.80 (0.28–2.25)	1.53 (0.55–4.29)	0.556

Abbreviations: CRC, colorectal cancer; HR, hazard ratio; CI, confidence interval. ^a^ Events are defined as all-cause deaths and CRC-specific deaths for CRC survivors. ^b^ Cox proportional hazard model adjusted for age at diagnosis, sex, stage at diagnosis, marital status, microsatellite instable status, BRAF mutation status, chemoradiotherapy, and total energy intake where applicable. ^c^ Test for linear trend was based on the median values for each quartile of intake.

**Table 3:** Characteristics of prospective cohort studies included in the meta-analysis of dietary fiber intake and outcome of CRC.

Author Year Country	Study Name	Study Population	Number of Cases	Follow-Up Time	Outcome	Exposure	Dose (Highest vs. Lowest Categories)	HR (95% CI)	Adjusted Variables
Dray [12] 2003 France	Influence of dietary factors on colorectal cancer survival	148 participants	46 deaths	5 years	5-year survival rate	Fiber	No dose	1.87 (0.83–4.22)	Age, sex, tumor stage, tumor location, and energy intake
Song [11] 2018 US	Fiber intake and survival after colorectal cancer diagnosis	1575 participants	773 deaths; 174 deaths from CRC	8 years (median)	CRC-specific mortality	Fiber	28.9 vs. 14.4 g/day	0.54 (0.35–0.85)	Age at diagnosis, sex, cancer stage, year of diagnosis, tumor grade of differentiation, subsite, fiber intake, post-diagnostic alcohol consumption, pack-years of smoking, BMI, physical activity, regular use of aspirin, glycemic load, and consumption of total fat, folate, calcium, and vitamin D
					All-cause mortality		28.9 vs. 14.4 g/day	0.64 (0.51–0.80)	
Ward [13] 2016 Europe	Prediagnostic meat and fibre intakes in relation to colorectal cancer survival in the European Prospective Investigation into Cancer and Nutrition	3789 participants	1262 deaths; 1008 deaths from CRC	4.1 years (average)	CRC-specific mortality	Fiber	31.2 vs. 14.5 g/day	0.90 (0.69–1.17)	Age at diagnosis, sex, BMI, smoking status, tumor grade, tumor stage, year of tumor diagnosis, energy intake, Ca intake, folate intake, alcohol intake, and education
					All-cause mortality		31.2 vs. 14.5 g/day	0.84 (0.66–1.06)	
Zhao 2022 Canada	Association between dietary fiber intake and mortality among colorectal cancer survivors: results from the Newfoundland familial colorectal cancer cohort study and a meta-analysis of prospective studies	504 participants	159 deaths; 83 deaths from CRC	6.4 years (median)	CRC-specific mortality	Fiber	30.1 vs. 13.4 g/day (male)	0.60 (0.26–1.38) (male)	Age at diagnosis, sex, stage at diagnosis, marital status, microsatellite instable status, BRAF mutation status, chemoradiotherapy, and total energy intake
							31.1 vs. 14.5 g/day (female)	1.25 (0.31–5.08) (female)	
					All-cause mortality		30.1 vs. 13.4 g/day (male)	0.76 (0.43–1.37) (male)	
							31.1 vs. 14.5 g/day (female)	0.90 (0.34–2.29) (female)	

Abbreviations: CRC, colorectal cancer; HR, hazard ratio; CI, confidence interval; US, United States of America; BMI, body mass index.

## Data Availability

Data are available upon reasonable request.

## Supplementary Materials

The following supporting information can be downloaded at: https://www.mdpi.com/article/10.3390/cancers14153801/s1. Table S1: Search strategy; Table S2: Inclusion and exclusion criteria; Table S3: Newcastle–Ottawa quality assessment scale of the cohort studies included in meta-analysis; Table S4: Demographical and clinicopathological characteristics of included and excluded subjects; Figure S1: Summary funnel plot of studies examining the association between dietary intake and all-cause mortality as a test for publication bias; Figure S2: Summary Begg’s funnel plot and Egger’s publication bias plot of studies examining the association between dietary intake and mortality.
