# Supplementary material for: Association between Dietary Fiber Intake and Mortality among Colorectal Cancer Survivors: Results from the Newfoundland Familial Colorectal Cancer Cohort Study and a Meta-Analysis of Prospective Studies
Source: Cancers (Basel). 2022 Aug 4;14(15):3801. doi: 10.3390/cancers14153801 (PMC9367345; doi:10.3390/cancers14153801)
Supplement: Supplementary file 1 [file cancers-14-03801-s001.zip › cancers-1792067-supplementary.pdf]

cancers MDPI

# Association between dietary fiber intake and mortality among colorectal cancer survivors： results from the Newfoundland familial colorectal cancer cohort study and a meta-analysis of prospective studies

Jing Zhao [1,5], Yun Zhu [1,5], Meizhi Du [1], Yu Wang [1], Jillian Vallis [2], Patrick S. Parfrey [3], John R. Mclaughlin [4], Xiuying Qi [1,*] and Peizhong Peter Wang [2,4,*]

**Table S1.** Search strategy.

| Set | Item | Field ( PubMed, Cochrane Library ) | Field (EMBASE ) |
|---|---|---|---|
| 1 | Dietary fiber | [Mesh] | [Emtree] |
| 2 | Food | [Mesh] | [Emtree] |
| 3 | Diet | [Mesh] | [Emtree] |
| 4 | Sets 1-3 were combined with “OR” | [Mesh] | [Emtree] |
| 5 | Survival | [Mesh] | [Emtree] |
| 6 | Mortality | [Mesh] | [Emtree] |
| 7 | Durvival rate | [Mesh] | [Emtree] |
| 8 | Death | [Mesh] | [Emtree] |
| 9 | Prognosis | [Mesh] | [Emtree] |
| 10 | Sets 5-9 were combined with “OR” | [Mesh] | [Emtree] |
| 11 | Colon | [Mesh] | [Emtree] |
| 12 | Large intestine | [Mesh] | [Emtree] |
| 13 | Rectum | [Mesh] | [Emtree] |
| 14 | Sets 11-13 were combined with “OR” | [Mesh] | [Emtree] |
| 15 | Neoplasm | [Mesh] | [Emtree] |
| 16 | Sets 14-15 were combined with “AND” | [Mesh] | [Emtree] |
| 17 | Colorectal tumor | [Mesh] | [Emtree] |
| 18 | CRC | [Mesh] | [Emtree] |
| 19 | Sets 16,17,18 were combined with “OR” | [Mesh] | [Emtree] |
| 20 | Sets 4,10,19 were combined with “AND” | [Mesh] | [Emtree] |

**Table S2.** Inclusion and exclusion criteria for the meta-analysis

| Factors | Inclusion criteria | Exclusion criteria |
|---|---|---|
| Study design | Prospective cohort study | In vitro, animal, or experimental study; clinical trials; ecological studies; review or meta-analyses |
| Setting | Full-text of original articles | Abstract ,editorials, communication or letter |
| Participants | CRC patients | People other than CRC patients |
| Methodology | HR (95% CIs) | Insufficient data |
| Exposure | Dietary fiber intake | Unrelated exposure |
| Outcome | All-cause mortality, CRC-specific mortality | Unrelated outcome |

Abbreviations: CRC, colorectal cancer; HR, hazard ratio; CIs, confidence intervals.

For dose-response meta-analysis, the eligibility criteria for included studies were as followed:

- At least three categories of dietary fiber intake;
- Hazard ratios (HR) estimates and 95% confidence intervals (CIs) for each category of dietary fiber intake levels were available or calculable;
- Category-specific or a total number of cases, and the category-specific or the total number of non-cases or person-years were provided. Comparability

**Table S3.** Newcastle-Ottawa quality assessment scale of the cohort studies included in the meta-analysis.

| Author year | Selection | | | | Comparability | Outcome/Exposure | | | Total score |
|---|---|---|---|---|---|---|---|---|---|
| | Item 1 | Item 2 | Item 3 | Item 4 | Item 5 | Item 6 | Item 7 | Item 8 | |
| Dray 2003 | - | 1 | - | 1 | 2 | - | 1 | 1 | 6 |
| Song 2018 | - | - | 1 | 1 | 2 | 1 | 1 | 1 | 7 |
| Ward 2016 | - | 1 | - | 1 | 2 | 1 | 1 | 1 | 7 |
| Zhao 2022 | - | - | 1 | 1 | 2 | 1 | 1 | 1 | 7 |

**Table S4.** Demographical and clinicopathological characteristics of the included and excluded subjects.

| Characteristics | Subjects included in this study (*n*=504) | | Subjects excluded due to lack of diet/disease-outcome data or unreliable total caloric intake (*n*=233) |
|---|---|---|---|
| | No. of survived patients | No. of deaths (%) | No. of patients (%) |
| Age at diagnosis (y) a | 60.3±9.0 | 62.0±8.9 | 61.7±10.1 |
| Sex | | | |
| Male | 200 (58.0) | 106 (66.7) | 143 (61.4) |
| Female | 145 (42.0) | 53 (33.3) | 90 (38.6) |
| BMI (kg/m$^2$) | | | |
| <25.0 | 97 (29.0) | 43 (27.9) | 59 (35.5) |
| 25.0-29.9 | 133 (39.7) | 70 (45.5) | 64 (38.6) |
| ⩾30 | 105 (31.3) | 41 (26.6) | 43 (25.9) |
| Marital status | | | |
| Single | 69 (20.0) | 40 (25.2) | 43 (24.0) |
| Married or living as married | 276 (80.0) | 119 (74.8) | 136 (76.0) |
| Tumor location | | | |
| Colon | 231 (67.0) | 97 (61.0) | 133 (72.7) |
| Rectum | 114 (33.0) | 62 (39.0) | 50 (27.3) |
| Stage at diagnosis | | | |
| I/II | 227 (65.8) | 66 (41.5) | 64 (27.5) |
| III/IV | 118 (34.2) | 93 (58.5) | 169 (82.5) |
| T stage | | | |
| T1 | 20 (6.5) | 5 (3.5) | 10 (4.9) |
| T2 | 77 (25.0) | 23 (16.1) | 15 (7.3) |
| T3 | 200 (64.9) | 107 (74.8) | 152 (73.8) |
| T4 | 11 (3.6) | 8(5.6) | 29 (14.1) |

**Table S4.** *cont.*

| Characteristics | Subjects included in this study (*n*=504) | | Subjects excluded due to lack of diet/disease-outcome data or unreliable total caloric intake (*n*=233) |
|---|---|---|---|
| | No. of survived patients | No. of deaths (%) | No. of patients (%) |
| N stage | | | |
| NX | 7 (2.3) | 2 (1.4) | 6 (2.9) |
| N0 | 197 (64.2) | 66 (46.8) | 62 (30.2) |
| N1 | 78 (25.4) | 43 (30.5) | 67 (32.7) |
| N2 | 25 (8.1) | 30 (35.5) | 70 (34.2) |
| M stage | | | |
| MX | 164 (58.0) | 56 (43.1) | 61 (33.1) |
| M0 | 111 (39.2) | 43 (33.1) | 57 (31.0) |
| M1 | 8 (2.8) | 31 (23.9) | 66 (35.9) |
| Chemoradiotherapy | | | |
| No | 62 (18.0) | 38 (23.9) | 38 (20.8) |
| Yes | 283 (82.0) | 121 (76.1) | 145 (79.2) |
| MSI status | | | |
| MSS/MSI-L | 277 (84.5) | 146 (97.3) | 201 (92.2) |
| MSI-H | 51 (15.6) | 4 (2.7) | 17 (7.8) |
| Smoking status | | | |
| Never smokers | 102 (29.6) | 36 (22.6) | 58 (33.5) |
| Ever smokers | 243 (70.4) | 123 (77.4) | 115 (66.5) |
| Total energy intake (kcal/d) a | 2439.3±874.4 | 2491.5±796.7 | 4462.0±3230.1 |

Abbreviations: BMI, body mass index; MSI, microsatellite instability; MSI-H, microsatellite instability-high; MSS/MSI-L, microsatellite stable/microsatellite instability-low; HR, hazard ratio; CI, confidence interval.
[a] Continuous variables presented as mean ± SD (standard deviation).
For some variables, totals may not add up due to missing values.

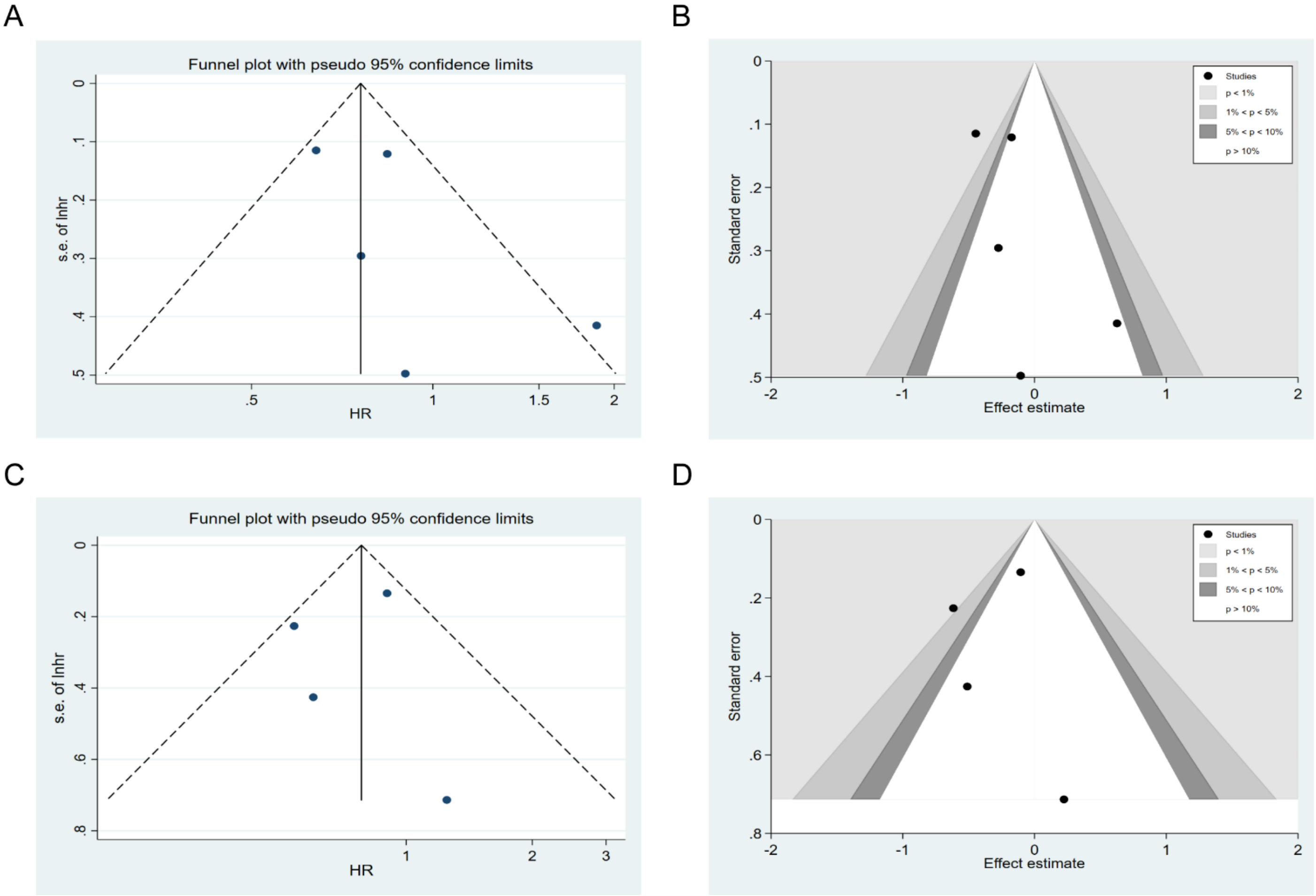


**Figure S1.** Summary funnel plot of studies examining the association between dietary intake and all-cause mortality as a test for publication bias. (**A**) Funnel plot for all-cause mortality; (**B**) Contour-enhanced funnel plot for all-cause mortality; (**C**) Funnel plot for CRC-specific mortality; (**D**) Contour-enhanced funnel plot for CRC-specific mortality.

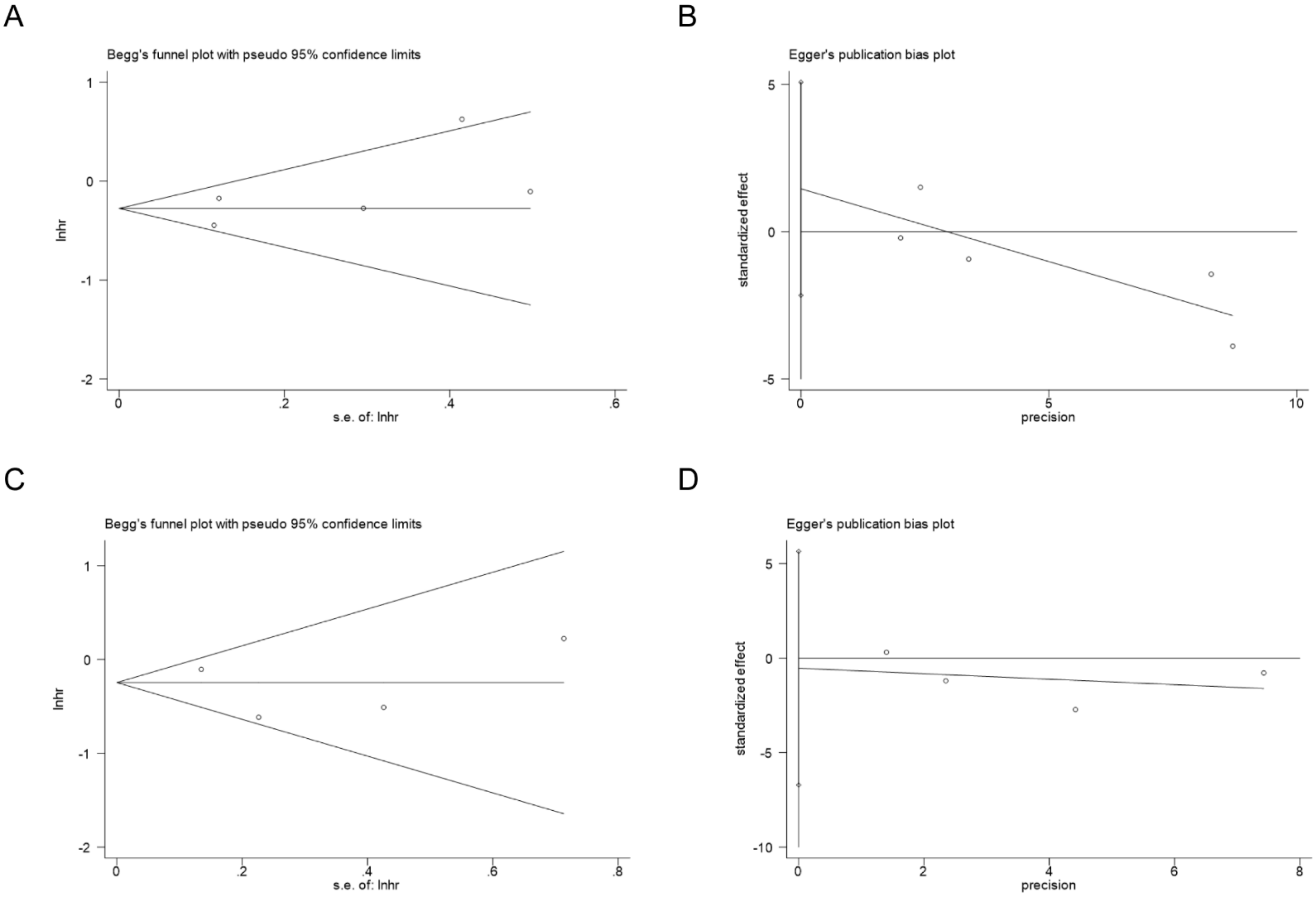


**Figure S2.** Summary Begg's funnel plot and Egger's publication bias plot of studies examining the association between dietary intake and mortality. (**A**) Begg's funnel plot for all-cause mortality; (**B**) Egger's publication bias plot for all-cause mortality; (**C**) Begg's funnel plot for CRC-specific mortality; (**D**) Egger's publication bias plot for CRC-specific mortality.
